# Hypnosis, Meditation, and Self-Induced Cognitive Trance to Improve Post-treatment Oncological Patients’ Quality of Life: Study Protocol

**DOI:** 10.3389/fpsyg.2022.807741

**Published:** 2022-02-10

**Authors:** Charlotte Grégoire, Nolwenn Marie, Corine Sombrun, Marie-Elisabeth Faymonville, Ilios Kotsou, Valérie van Nitsen, Sybille de Ribaucourt, Guy Jerusalem, Steven Laureys, Audrey Vanhaudenhuyse, Olivia Gosseries

**Affiliations:** ^1^Sensation and Perception Research Group, GIGA Consciousness, University of Liège, Liège, Belgium; ^2^Coma Science Group, GIGA Consciousness, University of Liège, Liège, Belgium; ^3^Trance Science Research Institute, Paris, France; ^4^Arsène Burny Cancerology Institute, CHU Liège, Liège, Belgium; ^5^Faculty of Psychology and Educational Sciences, Free University of Brussels and Emergences Association, Brussels, Belgium; ^6^Medical Oncology Department, CHU Liège and University of Liège, Liège, Belgium; ^7^Centre du Cerveau^2^, CHU Liège, Liège, Belgium; ^8^Algology Interdisciplinary Center, CHU Liège, Liège, Belgium

**Keywords:** oncology, group intervention, hypnosis, meditation, self-induced cognitive trance, neurophysiology, neurophenomenology

## Abstract

**Introduction:**

A symptom cluster is very common among oncological patients: cancer-related fatigue (CRF), emotional distress, sleep difficulties, pain, and cognitive difficulties. Clinical applications of interventions based on non-ordinary states of consciousness, mostly hypnosis and meditation, are starting to be investigated in oncology settings. They revealed encouraging results in terms of improvements of these symptoms. However, these studies often focused on breast cancer patients, with methodological limitations (e.g., small sample size, no control group, and no follow-up). Another non-ordinary state of consciousness may also have therapeutic applications in oncology: self-induced cognitive trance (SICT). It seems to differ from hypnosis and meditation, as it involves the body more directly. Thus, investigating its clinical applications, along with hypnosis and meditation interventions, could improve available therapeutic options in oncology. This article details the study protocol of a preference-based longitudinal controlled superiority trial aiming to assess the effectiveness of 3 group interventions (hypnosis, meditation, and SICT) to improve oncological patients’ quality of life, and more specifically CRF, emotional distress, sleep, pain, and cognitive difficulties (primary outcomes).

**Methods and analysis:**

A power analysis required a total sample of 160 patients. Main inclusion criteria are: cancer diagnosis, active treatments completed for less than a year, no practice of hypnosis, meditation, or SICT, and presence of at least one of these four symptoms: fatigue, sleep difficulties, depression, or anxiety. Each participant will choose the intervention in which they want to participate (hypnosis, mindful self-compassion meditation, SICT, or no intervention—control group). To test the effectiveness of the interventions, data will be collected by questionnaires and neurobiological measures and directly from the medical record at four time points: before inclusion in the study (baseline); immediately after the intervention; and at 3- and 12-month follow-up. The longitudinal data in each group will then be measured.

**Discussion:**

In addition to standard cancer therapies, there is a growing interest from patients in complementary approaches, such as hypnosis, meditation, and SICT. The results of this study will be useful to increase knowledge about short- and long-term effectiveness of 3 group interventions for CRF, emotional distress, sleep, pain, and cognitive difficulties in patients with different cancers.

**Clinical Trial Registration:**

ClinicalTrials.gov/ (NCT04873661). Retrospectively registered on the 29th of April 2021. url: https://clinicaltrials.gov/ct2/show/NCT04873661

## Introduction

The presence of one symptom cluster is particularly well-documented among patients who suffer from cancer (Dodd et al., 2010; Die Trill, 2013; Loh et al., 2018): cancer-related fatigue (CRF), sleep difficulties, emotional distress, and pain. Cognitive impairments are also frequently associated with this symptom cluster (Pullens et al., 2010; Joly et al., 2011; Sanford et al., 2014). A meta-analysis revealed that 52% of patients report CRF, this proportion ranging from 14 to 100% according to the studies (Ma et al., 2020). CRF can be defined as “a distressing persistent, subjective sense of physical, emotional, and/or cognitive tiredness related to cancer or cancer treatment that is not proportional to recent activity and interferes with usual functioning” (Koh et al., 2019). CRF has a lot of social, financial, and functional consequences (Jones et al., 2016; Ma et al., 2020). Emotional distress, endured by a large proportion of patients with cancer as well (Mehnert et al., 2018; Götze et al., 2020), can be defined as “a multifactorial, unpleasant experience of a psychologic (i.e., cognitive, behavioral, and emotional), social, spiritual, and/or physical nature that may interfere with the ability to cope effectively with cancer, its physical symptoms, and its treatment” (Mitchell et al., 2011; Riba et al., 2019). It negatively influences treatment adherence (Lin et al., 2017) and results (Batty et al., 2017), as well as patient’s general quality of life (Achimas-Cadariu et al., 2015; Riba et al., 2019). Concerning sleep difficulties, their prevalence among patients with cancer during or after their treatment varies between 32 and 61% (Santoso et al., 2019; Schieber et al., 2019; Hoang et al., 2020; Gonzalez et al., 2021) and is approximately three times higher than in the general population (Kwak et al., 2020). Cancer-related pain is reported by more than 50% of patients, which severely impacts quality of life, adherence to treatment, satisfaction with care, and survival (Neufeld et al., 2017). Finally, cognitive difficulties may be linked to the disease itself, the treatments received or the patient characteristics, and their prevalence can be up to 75% in some kinds of cancers (Joly et al., 2011).

All these difficulties can last for years after treatment completion and, despite their prevalence and severe impact, they remain underdiagnosed and undertreated (Joly et al., 2011; Die Trill, 2013; de Vries and Stiefel, 2014; Riba et al., 2019). In oncology settings, there is a growing interest in complementary approaches, such as “mind–body” interventions, to relieve these symptoms in a non-pharmacological way. These interventions aim to impact two interconnected levels: psychological and physical (Sawni and Breuner, 2017), and they allow patients to take back the control over their health and to regain hope (Eliott et al., 2008). Indeed, complementary and alternative medicine is estimated to be used by 43% of patients with cancer (Horneber et al., 2012; John et al., 2016), and it is starting to be studied scientifically, for instance with trials investigating the benefits of hypnosis and meditation (Spiegel, 1985; Kim et al., 2013; Cramer et al., 2015; Carlson et al., 2017; Grégoire et al., 2017, 2020a; Arring et al., 2019; Ngamkham et al., 2019; Suh et al., 2021).

### Hypnosis

**Hypnosis** can be defined as “a state of consciousness involving focused attention and reduced peripheral awareness characterized by an enhanced capacity for response to suggestion” (Elkins et al., 2015). It is characterized by four main components: absorption (in an imaginative experience), dissociation (from the environment), suggestibility (to the suggestions made by the therapist; Spiegel, 1991), and automaticity (non-voluntary response relevant to the content of a communication intended to be a suggestion; Weitzenhoffer, 2002). Hypnosis modulates self-awareness and decreases environmental awareness (Demertzi et al., 2011, 2015). At a neurophysiological level, it is associated with increased power in the theta band and changes in gamma activity (direction inconsistent among studies), which play a critical role in the emotional limbic circuits (Vanhaudenhuyse et al., 2020). Hypnosis also modifies the functional connectivity in the default mode network (linked to self-awareness) and in the external network (linked to the environmental awareness; Vanhaudenhuyse et al., 2014), as well as the structural connectivity between brain areas, and the hemispheric asymmetry (Landry et al., 2017). It also impacts cortical and subcortical areas involved in pain modulation (Vanhaudenhuyse et al., 2014; De Benedittis, 2021; Bicego et al., 2021c).

To summarize empirical findings about hypnosis mechanisms, Jensen et al. proposed a biopsychosocial model of hypnosis (Jensen et al., 2015). Three categories of factors explained hypnotic responding: biological factors (activity in frontal and anterior cingulate cortices, functional connectivity, structural connectivity, hemisphere asymmetry, and theta band), psychological factors (expectancies, hypnotizability, motivation, and absorption), and social factors (relationship with the therapist and hypnotic context). Several studies have demonstrated the benefits of hypnosis, used alone or in combination with other techniques, on different sides effects of cancer treatments, such as CRF, emotional distress, sleep, pain, and cognitive difficulties (Spiegel, 1985; Cramer et al., 2015; Carlson et al., 2017; Grégoire et al., 2017, 2020a; Arring et al., 2019).

### Meditation

Similarly to hypnosis, **meditation** is also considered a non-ordinary state of consciousness. It can be defined as a group of “states, processes, and practices that self-regulate the body and mind, thereby affecting mental and physical events by engaging a specific attentional set” (De Benedittis, 2015). Its aim is to improve voluntary control over mental processes. Different meditation practices exist, with similarities: a quiet location with few distractions, a specific and comfortable posture (sitting or lying down), a focus on attention, and an open attitude of letting thoughts come and go without judgment (Ospina et al., 2008). Phenomenological characteristics of meditation vary according to the type of practice, but alteration of sense of time, space and body representation, along with modifications of emotions and physical sensations are commonly reported (Brandmeyer et al., 2019). The neurophysiological correlates of mediation also depend on the type of meditation and thus are not yet clear (De Benedittis, 2015; Lomas, 2015; Brandmeyer et al., 2019). However, a systematic review showed that mindfulness-based meditation is associated with increased alpha and theta power, while no consistent patterns were observed in beta, delta, and gamma bandwidths. This configuration is indicative of a state of relaxed alertness favoring mental health (Lomas, 2015; Brandmeyer et al., 2019). Meditation has also been shown to positively influence emotional distress, pain, fatigue, and sleep difficulties in patients with cancer (Kim et al., 2013; Carlson et al., 2017; Arring et al., 2019; Ngamkham et al., 2019; Suh et al., 2021). Our study will focus on mindful self-compassion (MSC) meditation (Kotsou and Heeren, 2011; Germer and Neff, 2013, 2019; Neff and Germer, 2013; Neff et al., 2020), which involves a compassionate stance toward oneself when encountering personal difficulties (i.e., self-kindness over self-judgment, sense of common humanity instead of isolation, and mindfulness rather than over-identification), and has been shown to positively influence psychological wellbeing (Kotsou and Leys, 2016; Kılıç et al., 2021). This self-compassionate frame of mind involves being gentle, supportive, accepting, and understanding toward oneself (Germer and Neff, 2019).

Most of these studies about hypnosis and meditation suffer from methodological limitations, the main one being their focus on breast cancer patients only. However, other cancers may have different negative physical and psychological effects. For example, mortality rates vary according to the localization of the tumor (Sung et al., 2021). Additionally, some cancers imply specific adverse events [e.g., prostate cancers (impact on sexuality, possible urinary incontinence) and head and neck cancers (possible tracheotomy with impact on speech)], which may have specific impact on the psychological adaptation to the disease. In addition, most of these studies did not measure the long-term effects of the intervention proposed.

In this study, our aim will be to include a large population of patients with different cancer diagnoses and to rigorously investigate the long-term effects of the interventions. We also want to study another non-ordinary state of consciousness, involving more directly the body: self-induced cognitive trance.

### Self-Induced Cognitive Trance

The recent work on hypnosis and meditation has opened the path to study other non-ordinary states of consciousness, such as trance. Self-induced cognitive trance (SICT) is characterized by lucid but narrowed awareness of the environment, hyper-focused immersive experience of flow, enhanced inner imagery, modified somatosensory processing, altered sense of self, and an experience of spiritual travel (Csikszentmihalyi, 1990; Flor-Henry et al., 2017; Grégoire et al., 2021). It is inherited from Mongolian traditional shamanic practice, where is it used in healing interventions (Flor-Henry et al., 2017; Grégoire et al., 2021). However, little is known about the scientifically based phenomenology and neural correlates of SICT. A few case studies report that it is associated with subjective changes in time and space perception, body awareness, thinking and emotional state, with a dissociation and a modulation of perceptions from the environment, as well as decreased pain perception, increased strength, increased sense of happiness, visual imagery, and out-of-body experience (Hove et al., 2016; Flor-Henry et al., 2017; Kawai et al., 2017; Mainieri et al., 2017; Gosseries et al., 2020; Huels et al., 2021). This could be put in parallel with some of the characteristics of hypnosis already described (absorption, dissociation, and modulation of pain, for examples; Vanhaudenhuyse et al., 2014, 2020). Only a few studies used neurophysiological measurements during different kinds of trance, including SICT (Oohashi et al., 2002; Peres et al., 2012; Hove et al., 2016; Flor-Henry et al., 2017; Gosseries et al., 2020), and showed a temporary reconfiguration of brain network architecture. However, results are quite contradictory, and studies are mostly from small samples of trance experts with a lack of well-controlled designs. Moreover, no study has evaluated the clinical applications of SICT, even though it may have potential therapeutic properties, like hypnosis and meditation. Previous works on shamanic trance have reported positive outcomes (e.g., decrease anxiety and increase wellbeing) anecdotally, but not scientifically (Sidky, 2009; Mackinnon, 2012; Grégoire et al., 2021). Thus, investigating SICT’s phenomenological and neurophysiological correlates, as well as its clinical applications, and comparing them with hypnosis and meditation interventions seems particularly relevant as it will allow to better understand these non-ordinary states of consciousness and improve available complementary therapeutic options in oncology.

## Objectives

This project’s aims are threefold: (1) Evaluating the short- and long-term clinical benefits of hypnosis, meditation, and SICT in terms of CRF, emotional distress, sleep difficulties, and pain (primary outcomes), as well as other psychological variables (e.g., cognitive functioning, adaptation to cancer, psychological flexibility; secondary outcomes) in patients with cancer (through questionnaires); (2) Measuring the evolution of (a) phenomenological and (b) neurobiological correlates of hypnosis, meditation, and SICT in these patients, to better understand the effects of these interventions (through questionnaires and neurobiological measures); (3) Confirming the biopsychosocial model of hypnosis and investigating whether meditation and SICT responsiveness are mediated by the same mechanisms as hypnosis. This study will allow to strengthen the use of hypnosis and meditation for all cancers and to assess the interest of SICT as a new therapeutic complementary option.

## Methods and Analysis

### Design

**Figure 1:**
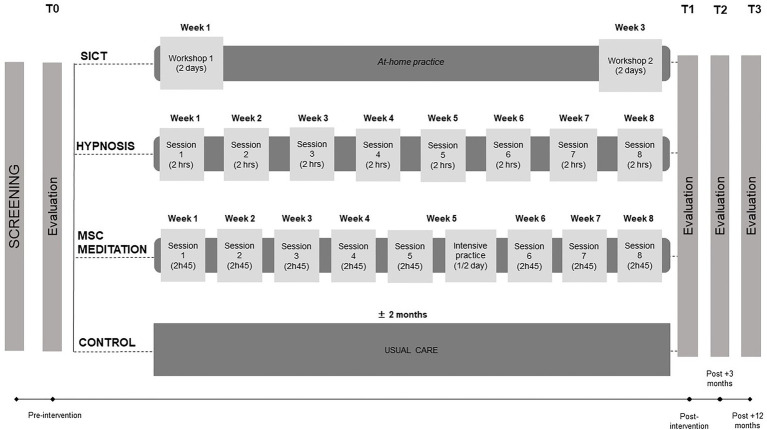
Study design. SICT, self-induced cognitive trance; MSC, mindful self-compassion; hrs, hours.

We designed a longitudinal controlled superiority trial (see Figure 1) with 160 patients with cancer who will choose between four conditions (see section “Recruitment” for sample size calculation): hypnosis-based group intervention, MSC meditation-based group intervention, SICT-based group intervention, or a control group. This kind of design has been chosen because patients who receive their preferred therapeutic option seem to be more motivated and exhibit greater adherence to the treatment (King et al., 2005; Preference Collaborative Review Group, 2008; Sedgwick, 2013; Bicego et al., 2021a). Indeed, preference-based trials are increasingly developed as researchers often want to know if an intervention is effective for the participants who choose it, rather than determining the best treatment option irrespective of the participant’s choice (Kowalski and Mrdjenovich, 2013). In addition, pragmatic trials are also more and more developed and aim at assessing the effects of interventions in real-life routine practice conditions, rather than under optimal conditions. The results from a pragmatic trial can be generalized and applied in routine practice (Patsopoulos, 2011). Moreover, one of our previous study with oncological patients showed that different intervention groups, based on participants’ preferences, were similar at baseline on the sociodemographic, medical, and clinical data investigated (Grégoire et al., 2017). Participants will then know in which group they belong and will be aware of the existence of the other intervention groups.

The structure and intensity of each intervention will be different (see Figure 1), because we want to optimize the trainings in a manner that has been shown to be effective in our practice with volunteers and patients (Grégoire et al., 2017, 2018b). More specifically, in the hypnosis-based intervention, patients will participate in 8 weekly sessions (2 h each) during which they will benefit from guided hypnosis exercises and learn how to implement self-hypnosis in their daily life. In the MSC meditation-based intervention, patients will participate in 8 weekly sessions (2 h45 each) and half a day of intensive practice, in which they will practice different meditation exercises. In the SICT-based intervention, patients will participate in 2-day workshops, spaced by 2 weeks, in which they will learn how to induce SICT, with the use of different sound loops (i.e., electronic designed sounds, voices, and noises). In the three groups, exercises will mostly focus on self-compassion as this component has been shown to be important in oncology settings in previous studies (Grégoire et al., 2017, 2018b, 2020a). At-home practice will also be encouraged between sessions and will be assessed through weekly evaluations (see Table 1 for measurements used). Participants in the control group will complete the questionnaires and undergo the neurobiological examinations, but they will not attend any of the interventions. They will only benefit from care as usual and have the possibility to participate in one of the three group interventions after the study completion if they want to. More information about the content of each intervention is detailed in section “Interventions”.

**Table 1:** Measurements used in the study.

	Screening	T0	Between T0 and T1 (during the group intervention)	T1	T2	T3
**Medical data**: diagnosis, time since the end of treatments, psychiatric and neurologic history	X					
**VAS**: fatigue, distress, pain, sleep difficulties (/10)	X					
**No practice** of hypnosis, meditation or SICT	X					
**Sociodemographic data**: age, gender, religious beliefs, professional status, BFI-10		X				
**Medical data**: time since diagnosis, cancer stage, treatment received		X				
**Weekly subjective evaluations** (VAS fatigue, emotional distress, sleep difficulties, pain)			X			
**Diary of practice** (frequency, details about some intense episodes, feelings, impressions, etc.)			X			
**Hypnosis/meditation/SICT and imaginary experience**: •VAS (/10): expectations, motivation to experience hypnosis/meditation/SICT^*^ •Personal definition and *a priori* according to the chosen intervention (free text)^*^ •CEQ		X				
**Quality of life-related variables**: •Fatigue and sleep: MFI20 and ISI •Emotional distress: HADS •Pain: VAS (/10) •Self-reported cognitive functioning: FACT-Cog v3 •Other psychological variables: MAC, CERQ, MPFI-24, Empowerment [7 VAS (/10)], major life events		X		X	X	X
**Neurophysiological and biological variables**: •EEG resting state		X		X		X
•EEG during hypnosis, meditation or SICT state^*^				X		X
•ECG, EMG, and respiration (effort and pressure)		X		X		X
•Core body temperature		X		X		X
•Tumor marker rates		X		X		X
**Questionnaires about the intervention**: •Free recall of a hypnosis/meditation/SICT/intense autobiographical episode (written, according to the intervention) •MCQ •MEQ30 •Presence questionnaire •VAS (/10): Quality of the relationship with the therapist^*^				X	X	X

BFI, Big Five Inventory; CEQ, Creative Experiences Questionnaire; CERQ, Cognitive Emotion Regulation Questionnaire; FACT-Cog, Functional Assessment of Cancer Therapy – Cognitive Function; HADS, Hospital Anxiety and Depression Scale; ISI, Insomnia Severity Index; MAC, Mental Adjustment to Cancer Scale; MCQ-30, Memory Characteristics Questionnaire; MEQ30, Revised Mystical Experience Questionnaire; MFI-20, Multidimensional Fatigue Inventory; MPFI-24, Multidimensional Psychological Flexibility Index; ECG, electrocardiogram; EEG, electroencephalogram; EMG, electromyogram; VAS, Visual Analogue Scale.

* Only for the participants in one of the 3 intervention groups.

Each participant from the three intervention groups will be evaluated before the intervention (T0), right after the last group session (T1), at 3 months (T2) and 1 year (T3) post-intervention. Participants in the control group will be evaluated according to a similar schedule (see Figure 1 for study design, and sections “Procedures” and “Assessments” for more information on procedures and measurements). The first intervention group (hypnosis) started in April 2021.

### Eligibility

Inclusion criteria will be: to be at least 18 years old, no psychiatric or neurological disorders, no current and regular practice of hypnosis, meditation or trance, diagnosis of cancer (any localization except brain tumors to avoid any effect on EEG data or severe cognitive impairments that could prevent filling the questionnaires adequately), and completion of active treatments (surgery, chemotherapy, radiotherapy) for less than a year. Interested patients will be screened for fatigue, emotional distress, sleep difficulties, and pain (VAS score ≥ 4/10 for one of these four symptoms for inclusion), based on recommendations from our past studies (Grégoire et al., 2018a,b).

### Recruitment

Participants will be recruited over a 2- to 3-year period, mainly at the University Hospital of Liège (Belgium) but also in other structures and through social medias. Potentially eligible participants will be identified in various ways. First, different health care professionals working with oncological patients (e.g., oncologists, radiotherapists, psychologists, nurses) have been informed of the study and asked to talk about it to their patients who have completed their treatment. In the hospital, interested patients will be advised to contact us by phone or e-mail. Second, flyers and posters will be displayed in several strategic areas in and outside the hospital (e.g., oncology, radiotherapy, algology services’ waiting rooms, doctors’ office, private consultations cabinet, other hospitals, health associations, health professionals in Belgium or France), which allow other health professionals and patients to be informed about our study. Third, we will use social medias (e.g., personal and professional Facebook accounts, Mewe) to inform as much people as possible about our study.

The recruitment started in December 2020 and is ongoing. Sample size has been determined by a power analysis allowing a comparison between the four groups. The sample size calculation was based on a repeated measures ANOVA (“between factors”). Alpha was set at 0.05, power at 90%, the standardized effect size at 0.3 and the expected correlation between repeated measures at 0.6. These coefficients were successfully used in our previous studies (Grégoire et al., 2017, 2020a). As there will be four groups and four measurements times, a total of 116 patients will be needed according to this analysis. Based on our previous experience with a similar design, we expect a 20% drop-out rate, leading to a sample of 140 patients (35 in each condition; Grégoire et al., 2020a). As our group sessions will include approximately 10 persons, we will then aim at recruiting 40 participants in each condition (i.e., four groups of 10 participants in hypnosis, MSC meditation, and SICT groups and 40 participants in the control group), for a final sample of 160 participants. Participants will have the opportunity to choose the intervention in which they want to participate. Most of the participants already have a strong preference for one of the three interventions when contacting us. However, each interested person who fill the inclusion criteria will be asked to watch a short video we made to explain the three interventions and the aims of the study.^1^ This allows every participant to receive standardized information about the study and to make or confirm their choice of intervention. However, once 40 participants have been recruited in a condition, it will not be possible for other interested persons to be included in this condition. These persons will be proposed to integrate the control group or another active condition.

### Procedures

A written consent will be obtained from each participant at the beginning of the study. The investigators will remain available by phone or e-mail during the study duration to answer any question.

#### Screening

During the first telephone contact with the interested participants, they will be informed of the protocol and study design. If interested, one of the two main investigators (CG or NM) will ensure that participants meet all the inclusion criteria. If eligible, they will have to watch the information video and confirm their choice of intervention. Once a group is almost complete (i.e., around 10 participants), the first evaluation (T0) will be scheduled.

#### Measurement Points (T0, T1, T2, and T3)

Each of these four measurement points will be completed by every participant and will consist of several questionnaires (at T0, T1, T2, and T3) and neurobiological measures (at T0, T1, and T3; see Table 1). Questionnaires will be completed online at home, on a secured website.^2^ For those who do not have a computer or are not comfortable using one, it will be possible to make an appointment with one of the investigators to complete the questionnaires with them. At each measurement point, the risk of missing responses is very low, as the online questionnaires are designed in a way that does not allow for missing answers. The investigators will check before the first group session that all participants have answered the questionnaires and remind them if it is not done. For the participants who will complete the questionnaires with one of the investigators, all answers will be checked to minimize missing data.

The neurophysiological and biological measures will be collected at the hospital (CHU of Liège), during a session of ±1 h (see point 3.6.). Tumor marker rates will be collected directly from the patient’s medical record or by asking them to send us their last blood test result. All data will be anonymized: a code will be attributed to each participant and used during the entire study. Only the researchers involved in this study will have access to the final datasets. Review of the trial process and difficulties encountered are discussed regularly between the researchers involved.

### Interventions

#### Hypnosis

This intervention will include eight 2h weekly sessions in groups of approximately 10 participants. This protocol has been developed and will be led by one of the authors (MEF), who is an anesthesiologist and an international expert in hypnosis. A large part of the first session will be devoted to answering to the participants’ questions and giving information about hypnosis. First exercises will be proposed as introduction to hypnosis: a focused listening of different musical records and some imagery exercises. The aim is for the participants to notice their abilities to access mental imagery and hypnosis. During the other sessions, a debriefing of at-home practice will be proposed, along with different hypnosis exercises. They will be repeated and discussed, and participants will also attempt to induce hypnosis by themselves during the last session. Table 2 summarizes the exercises that will be done at each session. Between sessions, participants will be encouraged to practice the different exercises at home with the help of audio recordings of the hypnosis exercises. This is essential to take full advantage of hypnosis without the help of a therapist. A self-care approach will be fostered during the intervention, more specifically during the debriefings of the participants’ practice, if they encounter difficulties or ruminations. Concrete strategies to deal with such difficulties will be proposed by the therapist. The importance to take time for themselves will be emphasized, as well as to allow themselves to take a break from their routine and practice hypnosis in their daily life to improve their wellbeing, in an adaptation of our usual practice with oncological patients in clinical and experimental settings (Charland-Verville et al., 2017; Grégoire et al., 2018b, 2020a).

**Table 2:** Summary of the content of the sessions for each intervention.

	Hypnosis	MSC meditation		Self-induced cognitive trance (SICT)
Session 1	Explanations about hypnosis and answers to questions. Exercises based on focused listening of musical records specially composed by a certified music therapist, and mental imagery.	Explanations about meditation and answers to questions. Exercises based on the discovery of mindful self-compassion (soothing touch, self-compassion break).	Workshop 1—Day 1	Explanations about SICT and answers to questions. SICT exercises using sound loops. Individual search of one or several movements or sounds to induce SICT without listening to the sound loops. Debriefing of each exercise in group. Focus on the transformative power of SICT (i.e., impact on bodily sensations and somatosensory processes, on cognition and emotions).
Session 2	Discussion of self-care strategies in accordance to the difficulties encountered by the participants. Soothing White Clouds exercises.	Mindful self-compassion exercises (affectionate breathing, grounding, mindfulness in daily life, present moment).		
Session 3	Discussion of self-care strategies in accordance to the difficulties encountered by the participants. Safe Place exercises.	Benevolent love exercises (affectionate breathing, love for a close one, self-compassion, and benevolent love).	Workshop 1—Day 2	SICT exercises using different sound loops and individual movements or sounds. Debriefing of each exercise in group. Focus on the transformative power of SICT.
Session 4	Discussion of self-care strategies in accordance to the difficulties encountered by the participants. Garden of dreams exercises.	Discovery of one’s compassionate voice exercises (self-compassion, security and self-criticism, self-compassionate letter).		
Session 5	Discussion of self-care strategies in accordance to the difficulties encountered by the participants. Pain and Colors exercises.	Importance of living intensely (giving and receiving compassion, personal values and wishes, silver lining, compassionate listening).	Workshop 2—Day 1	Debriefing of the 2-week individual practice. SICT exercises using different sound loops and individual movements or sounds. Debriefing of each exercise in group. Consolidation of the participants’ self-induction ability. Focus on the interactions during SICT (i.e., with the environment).
Between sessions 5 and 6	/	Half a day of silent intensive practice (review and deepening of all the exercises previously worked on)		
Session 6	Discussion of self-care strategies in accordance to the difficulties encountered by the participants. Glove Analgesia exercises.	Dealing with difficult emotions [self-compassion, acceptation and management of emotions, “soften, soothe, allow” (basic and specific to shame)].		
Session 7	Discussion of self-care strategies in accordance to the difficulties encountered by the participants. Levitation exercises.	Exploration of difficult relationships (compassionate friend, unfulfilled needs, Qi gong, self-compassion break in relationships, compassion with equanimity, dealing with compassion fatigue).	Workshop 2—Day 2	Debriefing of the 2-week individual practice. SICT exercises using different sound loops and individual movements or sounds. Debriefing of each exercise in group. Consolidation of the participants’ self-induction ability. Focus on the interactions during SICT.
Session 8	Discussion of self-care strategies in accordance to the difficulties encountered by the participants. Stories and Metaphors exercises.	Embracing life (self-compassion and compassion for others, appreciation and gratitude, self-compassion bracelets).		
Each session	Debriefing of the previous session and at-home practice.			

#### MSC Meditation

This intervention will include eight sessions of 2 h45 in groups of approximately 10 participants. It will be led by two of the authors (VvN and SdR), recognized as experts in MSC meditation. The intervention is based on the practice of MSC meditation, already described above. At the beginning of the first session, a presentation of the intervention will be proposed along with explanations about meditation and answers to participants’ questions. Each session will focus on a theme, linked to self-compassion (see Table 2), and will be based on formal practices (e.g., guided meditation), experiential exercises, informal practices linked to the daily life and theoretical information about compassion and emotions. Each exercise will be debriefed in small or large groups, in order to allow each participant to share their feelings, thoughts and questions. Between each session, individual practice will be encouraged and audio recordings of different exercises will be given to the participants.^3^ Between the fifth and the sixth session, half a day of silent intensive practice will be organized, during which all the exercises previously worked on will be reviewed and deepened.

#### Self-Induced Cognitive Trance

This intervention will include two 2-day workshops, spaced by two weeks, in groups of approximately 10 participants. A part of the first session will be devoted to explain what is SICT, how it can be accessed and how the workshops will take place. Possible questions among the participants will be discussed. To induce SICT, different sound loops (i.e., electronic designed sounds, voices, and noises, inspired by the ones produced during traditional shamanic rituals) will be used. They were designed and previously employed by one of the authors (CS), who is the first Westerner to become an *ugdan* (female shaman in Mongolia), and recognized as an international expert on SICT (Sombrun, 2006, 2012). Different kinds of trances will be taught during the workshops: trances with intention, during which participants follow an intention [e.g., refocusing on oneself (“anchor trance”), finding one’s place and being assertive (“territorial trance”), reconnecting with one’s inner power (“power trance”)], and trances without intention (“free trances”), during which participants let themselves and their body get carried away by the experience of trance. During each session, 4–5 SICT exercises will be proposed (see Table 2). Typically, participants will lie down and listen to one of the sound loops (± 30 min). The access to SICT can manifest itself through various elements: vocalizations or movements for examples. Every exercise will be debriefed in group, and a focus on self-compassion will be suggested during the workshops using specific intentions (e.g., an intention to be gentle with oneself, set self-boundaries). At the end of the two workshops, all participants will have found a way to induce SICT without the help of the sound loops. At-home practice will be encouraged during the two  weeks between the workshops and after them.

The three interventions will take place in Liège (hypnosis, SICT) and Brussels (MSC meditation). During the entire study duration, each participant will benefit from usual care, including medical care, oncological revalidation, and individual psychological help if necessary. Although no adverse event has been reported in our previous studies on hypnosis with patients with cancer or chronic pain (Grégoire et al., 2017, 2018b, 2020a; Bicego et al., 2021b) and clinical practice, it could be possible that a patient feels uncomfortable during the exercises. In this case, they will have the possibility to stop the session and their participation in the study. The therapist or the experimenter can also propose a meeting to discuss their difficulties and, if necessary, suggest a meeting with a psychologist or physician with whom we collaborate. Any reason for drop-out will be consigned. Adherence to the study is fostered by the facts that all participants are volunteers and motivated to participate and that the assessments can be mainly done at home and require only three travels to the hospital during the whole study. Several reminders will also be sent to the participants who forget to answer the questionnaires in time and who did not ask to quit the study.

### Assessments

Table 1 summarizes the different parameters used at each measurement point.

#### Questionnaires

Approximate duration of questionnaires completion is 40–50 min at each measurement point.

##### General Information

Sociodemographic and medical data (gender, age, education level, professional activity, spiritual believes, personal history of cancer and treatment, possible recurrence during the study) will be collected. The Big Five Inventory (short version—10 items; Courtois et al., 2020) will also be administered. This questionnaire aims at rapidly assessing personality according to the 5 domains of the original Big Five Inventory (extraversion, conscientiousness, negative emotionality, open-mindedness, and agreeableness).

##### Physical and Psychological Functioning

- *Visual Analogue Scales (VAS)*: Different VAS will assess the participants’ emotional state (pain, empowerment, fatigue, emotional distress, sleep difficulties). VAS will also be used during the duration of the group intervention to assess the severity of fatigue, distress, sleep difficulties, and pain on a weekly basis. Each score will be comprised between 0 and 10.
- *Multidimensional Fatigue Inventory* (MFI-20; Smets et al., 1995): This 20-item scale is designed to measure fatigue on 5 different dimensions: general fatigue, physical fatigue, mental fatigue, reduced motivation, and reduced activity.
- *Insomnia Severity Index* (ISI; Savard et al., 2005): This 7-item questionnaire investigates the sleep complaints and the associated distress.
- *Hospital Anxiety and Depression Scale* (HADS; Zigmond and Snaith, 1983): This 14-item scale measures anxiety (7 items) and depression (7 items) and has been validated for people with somatic illnesses.
- *Mental Adjustment to Cancer Scale* (MAC; Watson et al., 1988): This questionnaire assesses the coping styles and adjustment to cancer. It is divided into two sub-scales: positive and negative adjustments.
- *Cognitive Emotion Regulation Questionnaire* (CERQ; Garnefski et al., 2001): This multidimensional scale investigates the cognitive emotion regulation strategies used after experiencing negative events linked to the disease or its treatments.
- *Multidimensional Psychological Flexibility Index* (MPFI-24; Grégoire et al., 2020b): This 24-item scale assesses the psychological flexibility and psychological inflexibility, through 12 dimensions (6 linked to psychological flexibility: acceptance, present moment awareness, self as context, defusion, contact with values, committed actions; and 6 for psychological inflexibility: experiential avoidance, lack of present moment awareness, self as content, fusion, lack of contact with values, inaction) and 2 total scores (psychological flexibility and inflexibility).
- *Life events*: To assess whether the participants experienced any major life event during or after the intervention, we will ask them the following question (yes/no): “*Did you experience any major life event since the last time you completed these questionnaires* (wedding, birth, job loss, accident, health problem, etc.).”

##### Cognitive Functioning

- *Functional Assessment of Cancer Therapy – Cognitive Function* (FACT-Cog v.3; Joly et al., 2012): This 20-item questionnaire measures the participant’s subjective cognitive functioning over the past week.

##### Questionnaires Linked to the Chosen Intervention (Hypnosis, Meditation or SICT)

- *VAS (/10)* concerning the expectations and motivation to participate in the intervention (T0), then concerning the quality of the relationship with the therapist (T1–T3).
- *Questions about the chosen intervention (free text)*: This will aim at investigating the personal definition of hypnosis, meditation, or SICT of participants, their beliefs and *a priori* concerning the intervention they will participate in.
- *Creative Experiences Questionnaire* (CEQ; Merckelbach et al., 2001): This 25-item (true/false) scale has been designed to investigate the fantasy proneness. Questions are about, for examples, imaginative friend or animal, belief in supernatural beings, tendency to dream of fantasize, or out-of-body experiences.
- *Free recall of an intense hypnosis/meditation/SICT episode (intense autobiographical episode for the participants in the control group)*: This will be used to collect phenomenological data about each non-ordinary state of consciousness involved in the study. As patients in the control group will not participate in any of the interventions, they will have to describe an important autobiographical memory that happened during the study (e.g., wedding, birth). The recall of the episode will have to be as detailed as possible.
- *Memory Characteristics Questionnaire* (MCQ; Johnson et al., 1988): This 16-item questionnaire is adapted from the original questionnaire. It assesses a wide range of memory characteristics (visual details, complexity, spatial, and temporal information, feelings). Participants will be asked to answer the questionnaire while thinking about one specific memory (the one used in the free recall).
- *Revised Mystical Experience Questionnaire* (MEQ30; Maclean et al., 2012): This 30-item scale is designed to assess the mystical characteristics of a specific episode or memory (in this study, a hypnosis, meditation, or SICT episode), across four factors: mystical, positive mood, transcendence of time/space, and ineffability.
- *Presence questionnaire* (Heck et al., 2021): This 12-item scale has been initially designed to measure the feeling of presence after the use of virtual reality. As the original questionnaire was focused on the virtual environment specific to virtual reality, we adapted it to a more general “suggested” environment, such as in hypnosis, meditation, and SICT. Questions are about the impression to have been present in the environment, to have interacted with it, or to have felt the presence of other people in it, for examples.

#### Neurophysiological and Biological Measures

We will record an electroencephalogram (EEG) resting state (256 EGI, Geodesics) during 15 min, combined (T1 and T3) or not (T0) with an EEG during hypnosis, meditation, or SICT state (15 min). During each EEG, we will also record an electrocardiogram (ECG) and an electromyogram (EMG), and measure the breathing (effort and pressure, Polygraph Input Box of EGI system) and body temperature. Tumor marker rates (according to the patient’s diagnosis) will also be collected in the patient’s medical record or based on their blood test results.

### Data Coding and Storage

Most questionnaires will be completed electronically (on www.alchemer.com) by the participants, through a secured link sent by the experimenters. Data encoding will be done automatically by Alchemer and will be regularly checked by the experimenters. Final databases and manually coded data (e.g., from manually completed questionnaires or directly collected from medical records) will be stored on a protected server from the University, protected by a password. Data coding and storage comply with the General Data Protection Regulation (GDPR).

### Statistical Analyses

Baseline (T0) demographic, medical, and psychological data will be compared between groups to test for initial groups equivalency using inferential statistics, including ANOVAs and chi-square tests. Normality of data will be checked using Shapiro–Wilk test. Group-by time changes, and pre- and post-assessment comparison of each variable within each group will be assessed using repeated measures MANOVA followed by post hoc comparisons, on the participants who completed all the needed assessments points. Hierarchical linear regressions will be conducted to investigate the factors associated with the evolution of our main outcomes. All tests will be two-tailed, and the alpha will be set at 0.05. For EEG data analysis, MATLAB, EEGLAB, FieldTrip, Brainstorm, and in-house MATLAB and Python modules will be used. EEG signal will be preprocessed as in our previous works [downsampling, epoching, filtering, removing noise (notably using ICA), channels interpolation, re-referencing; Carrière et al., 2020; Thibaut et al., 2021]. EEG markers will then be extracted: spectral (e.g., power spectrum density), connectivity (e.g., weighted symmetrical mutual information), complexity (e.g., permutation entropy; Engemann et al., 2018). We will also apply graph theory connectivity measures to further assess changes between brain regions (Chennu et al., 2017; Carrière et al., 2020; Thibaut et al., 2021). Results will be corrected for multiple comparisons and considered significant at *p* < 0.05. Statistical analyses will be performed after all data have been collected for T2, then for T3 and T4.

All participants will be informed by e-mail about the final results of the study. Scientific publications and presentations will also be planned.

## Discussion

Based on previous studies showing the benefits of different mind–body interventions, such as hypnosis and meditation, on cancer patients’ quality of life (Spiegel, 1985; Kim et al., 2013; Cramer et al., 2015; Carlson et al., 2017; Grégoire et al., 2017, 2020a; Ngamkham et al., 2019; Suh et al., 2021), and on the growing interest of these patients for such interventions, we designed a longitudinal controlled study assessing the benefits of three mind–body group interventions based on non-ordinary states of consciousness: hypnosis, MSC meditation, and SICT. Our aim is to investigate the short- and long-term effects of these interventions on physical and psychological symptoms of post-treatment oncological patients and to investigate their phenomenological experiences, their neurophysiological correlates, and their mechanisms of action. Our wish is also to include a variety of cancer diagnoses to address a major gap in the scientific literature: the lack of data about the effects of psychosocial interventions on cancers other than breast cancers.

Based on the existing scientific literature, our previous studies, and preliminary data (not published), we made several hypotheses:

1. **Hypnosis, MSC meditation, and SICT will have positive overall benefits on the quality of life of cancer patients**, with increased effects over time. More specifically, we expect that the three interventions will have superior positive effects on the patients’ quality of life than the control group. We expect that these effects will be significant right after the intervention (T1) and will stay stable or increase at 3- and 12-month follow-up (T2 and T3) due to continued practice. Moderate difference might be observed between the three intervention groups. For example, SICT could impact more the body-related variables (e.g., pain and fatigue) as it involves the body more directly (e.g., in the induction process). We expect that a small improvement will be achieved in the control group as well, but much weaker than in the three intervention groups.
2. **Phenomenological experiences will change over time and will be intervention-dependent**. We hypothesize that over time and in the three intervention groups, phenomenology will become more joyful and richer. As the phenomenology of hypnosis, MSC meditation, and SICT has been very little studied, we do not have any specific hypothesis concerning the phenomenological differences between the three interventions. Our analyses are exploratory. We however expect that the phenomenology will change in each intervention group between the different measurement times, as the participants are expected to continue to practice regularly after the intervention. We do not expect any major evolution of the phenomenological experiences in the control group.
3. **Neurophysiological changes will be different between the three intervention groups**. We hypothesize that neurophysiological changes will vary according to the intervention and that they will evolve over time, in response to the continued practice of the participants after the intervention. Previous studies showed increased theta power and changes in gamma activity during hypnosis (Vanhaudenhuyse et al., 2020) and increased alpha and theta power, during meditation (Lomas, 2015; Brandmeyer et al., 2019). We hypothesize to observe the same patterns in our study. We do not expect any evolution of neurophysiology in the control group. We also expect differences in neurophysiological correlates of resting state, depending on patients’ clinical outcomes, especially long-term ones. For example, we could expect slower activity if patients are feeling less anxious.
4. **We will be able to confirm, at least in part, the biopsychosocial model of hypnosis**, and MSC meditation and SICT responsiveness mechanisms will be in part similar to the ones implied in hypnosis responsiveness. We also expect other potential factors (linked to body physiology: temperature, ECG, EMG, breathing) to correlate with hypnosis, MSC meditation, and/or SICT responsiveness.

Participants are not aware of the specific hypotheses of our study. However, they have a general idea of the study aim, as the flyers and posters used for the recruitment underlined the fact that the purpose of each intervention is to improve their quality of life.

There are some limitations of our study. First, only patients who have finished their active treatments for less than a year can participate in the study. It is possible that patients who finished their treatments longer ago, or who are still in treatment, will be willing to participate. The same limitation applies for most of our inclusion criteria: some patients with brain tumor, or who are currently practicing hypnosis or meditation for example, could be interested in the study, and will not be able to participate. It is then possible that these inclusion criteria will impact the recruitment process and our results. However, they were chosen in order to minimize the baseline differences in our sample. Another limitation is linked to the profile of our participants. As our study takes place in Belgium, our participants will mainly come from Belgium or France or maybe French-speaking areas of other bordering countries. This means that all our participants will come from western and industrialized areas, which could induce some similarities among them. Our three interventions are also proposed in a group setting, which could discourage some people to participate as they could be uncomfortable in sharing their experience and thoughts with others. In addition, the therapists involved in our study could also be considered as a potential bias. Indeed, some of them are recognized as international experts in their discipline, and the fact that they will lead the group sessions is likely to impact the recruitment process, with more patients wanting to participate in their interventions. Finally, the design of our study could be considered as a limitation. First, our study will not be randomized, even though randomized-controlled trials are generally considered the gold standard for research aiming at assessing the effectiveness of an intervention (Hariton and Locascio, 2018). This preference-based design was chosen because we did not find it relevant for a patient who wants to participate, for example, in the hypnosis intervention to be obliged to participate in another intervention. Indeed, patients who can chose their treatment have more motivation and a greater adherence (King et al., 2005; Preference Collaborative Review Group, 2008; Sedgwick, 2013; Bicego et al., 2021a). We also chose this design based on our clinical observations. After a generally long cancer journey, where patients rarely had the opportunity to make any choice and had to endure several intense treatments and their side effects, it seemed important to us to allow them to finally take back the control over their care and choose the intervention they want to participate in. In addition, preference-based and pragmatic trials are more and more represented in scientific studies when the aim is to assess the effectiveness of an intervention for the participants who chose it, rather than determining the best treatment option (Kowalski and Mrdjenovich, 2013), or to assess the effectiveness of the intervention in real-life conditions (Patsopoulos, 2011). Second, the designs of the three interventions are different in terms of duration and frequency. As explained above, we decided to use these designs because they have been shown to be acceptable and effective in our practice with volunteers and patients. As the main aim of this study is not to investigate how each intervention allows an improvement of the patients’ quality of life, using different intervention designs seems understandable and acceptable. If our results are conclusive, future research should continue to investigate these three interventions and their mechanisms of action by standardizing their designs.

In conclusion, this project is particularly original, timely relevant and innovative. First, it will allow to adequately evaluate the short- and long-term effects of the interventions to improve the quality of life of a mixed population of patients who often suffer from intense and debilitating long-lasting cancer-related symptoms, along with behavioral, phenomenal and neurophysiological changes. This could lead to an improvement of mind–body interventions in oncology settings in the future, along with a better understanding of hypnosis, meditation, and SICT phenomena and their impact on quality of life. We could also imagine proposing these mind–body interventions to other clinical populations in the future (e.g., patients with chronic pain). Second, combining phenomenology and electrophysiology with rigorous methodology will be an asset to dive deep into the cognitive and brain functions related to hypnosis, meditation, and SICT, including their impact on physical and psychological symptoms.

## Ethics Statement

The studies involving human participants were reviewed and approved by Hospital-Faculty Ethics Committee of Liège. The patients/participants provided their written informed consent to participate in this study.

## Author Contributions

CG, AV, and OG participated in the conception and design of the study and in drafting the manuscript. CS, MEF, IK, and GJ participated in the conception and design of the study and in revising the manuscript critically for important intellectual content. NM, VvN, SdR, and SL participated in revising the manuscript critically for important intellectual content. CS, MEF, VvN, and SdR also led the intervention groups. All authors have read and approved the final manuscript and agreed to be accountable for all aspects of the work in ensuring that questions related to the accuracy or integrity of any part of the work are appropriately investigated and resolved.

## Funding

This study was funded by the Belgian National Funds for Scientific Research (FRS-FNRS)—Télévie (Grant 7460320), the Belgian Cancer Foundation (Grant C/2020/1357), the University of Liège and University Hospital of Liège, and the Benoit Foundation (Brussels—Belgium). These funds financed the different researchers involved in this study. The funding bodies have no role in the design of the study and collection, analysis, and interpretation of data and in writing the manuscript. CG is a postdoctoral researcher, OG is a Research Associate, and SL is a Research Director at the F.R.S-FNRS.

## Conflict of Interest

The authors declare that the research was conducted in the absence of any commercial or financial relationships that could be construed as a potential conflict of interest.

## Publisher’s Note

All claims expressed in this article are solely those of the authors and do not necessarily represent those of their affiliated organizations, or those of the publisher, the editors and the reviewers. Any product that may be evaluated in this article, or claim that may be made by its manufacturer, is not guaranteed or endorsed by the publisher.

## Footnotes

^1^ https://www.youtube.com/watch?v=lRhs8jK-RCE

^2^ www.alchemer.com

^3^ https://soundcloud.com/emergences/sets/cycle-auto-compassion

## Abbreviations

ANOVA: Analysis of variance
BFI: Big Five Inventory
CERQ: Cognitive Emotion Regulation Questionnaire
CEQ: Creative Experiences Questionnaire
CHU: “Centre hospitalier universitaire” (University Hospital)
CRF: Cancer-related fatigue
ECG: Electrocardiogram
EEG: Electroencephalogram
EMG: Electromyogram
FACT-Cog: Functional Assessment of Cancer Therapy - Cognitive Function
GDPR: General Data Protection Regulation
HADS: Hospital Anxiety and Depression Scale
Hrs: hours
ISI: Insomnia Severity Index
MAC: Mental adjustment to cancer
MCQ: Memory Characteristics Questionnaire
MEQ: Mystical Experience Questionnaire
MFI: Multidimensional fatigue inventory
MPFI: Multidimensional Psychological Flexibility Index
MSC: Mindful self-compassion
SICT: Self-induced cognitive trance
VAS: Visual analogue scale
